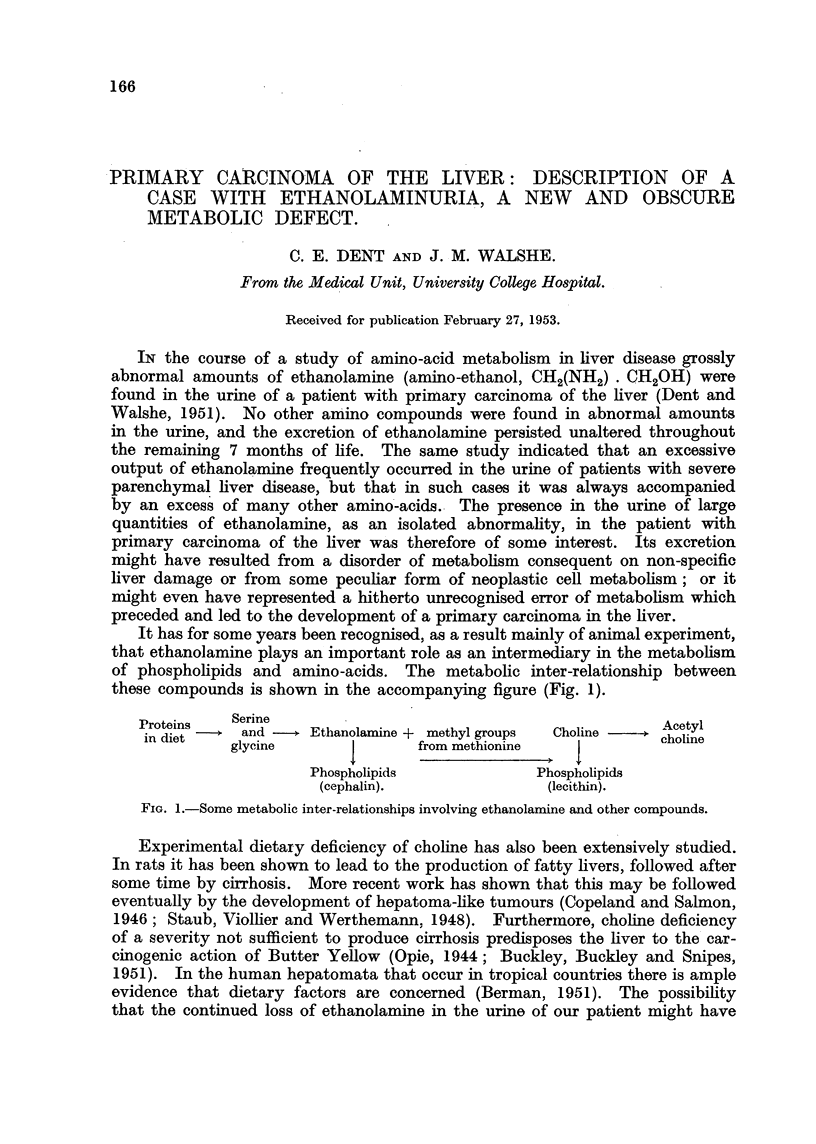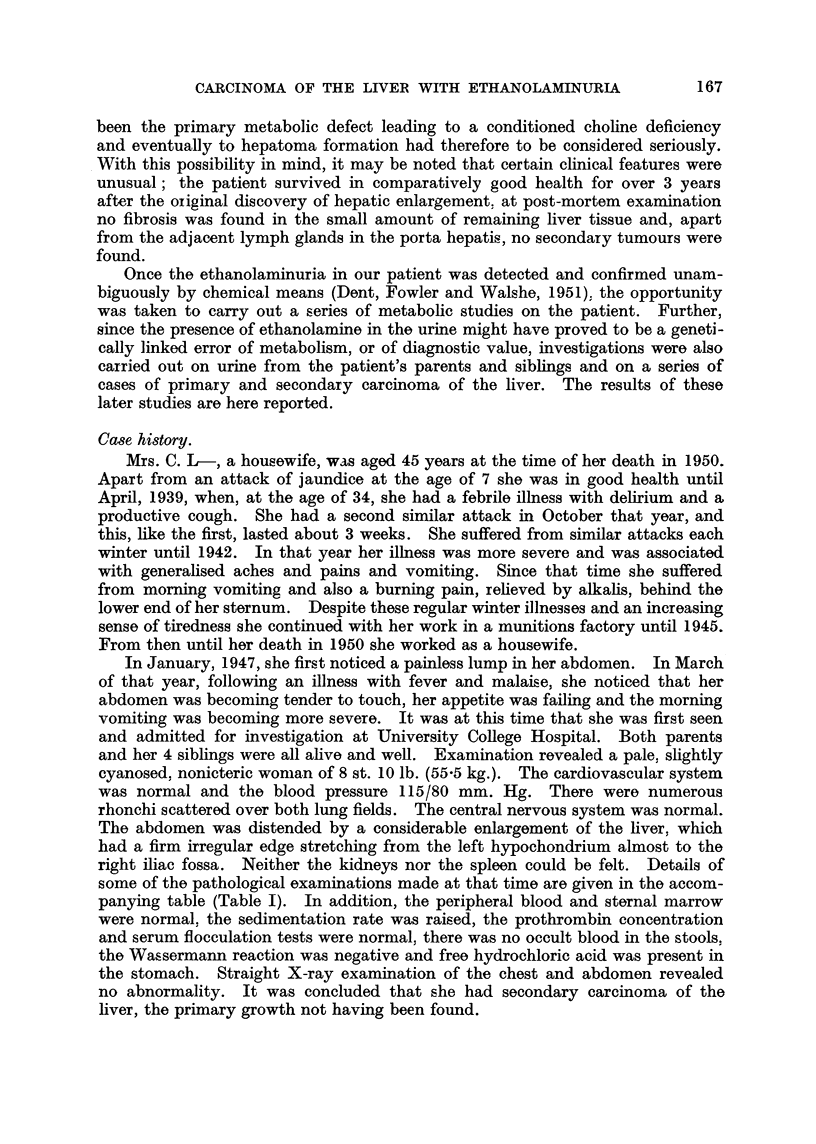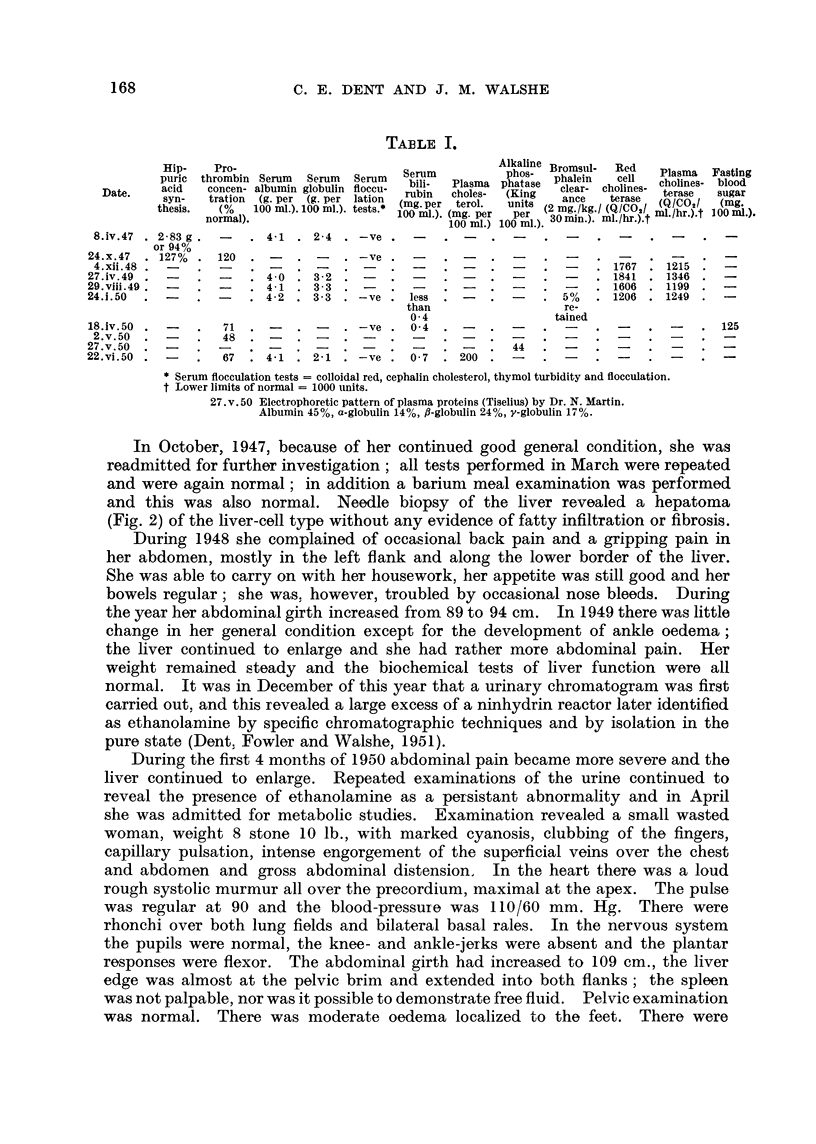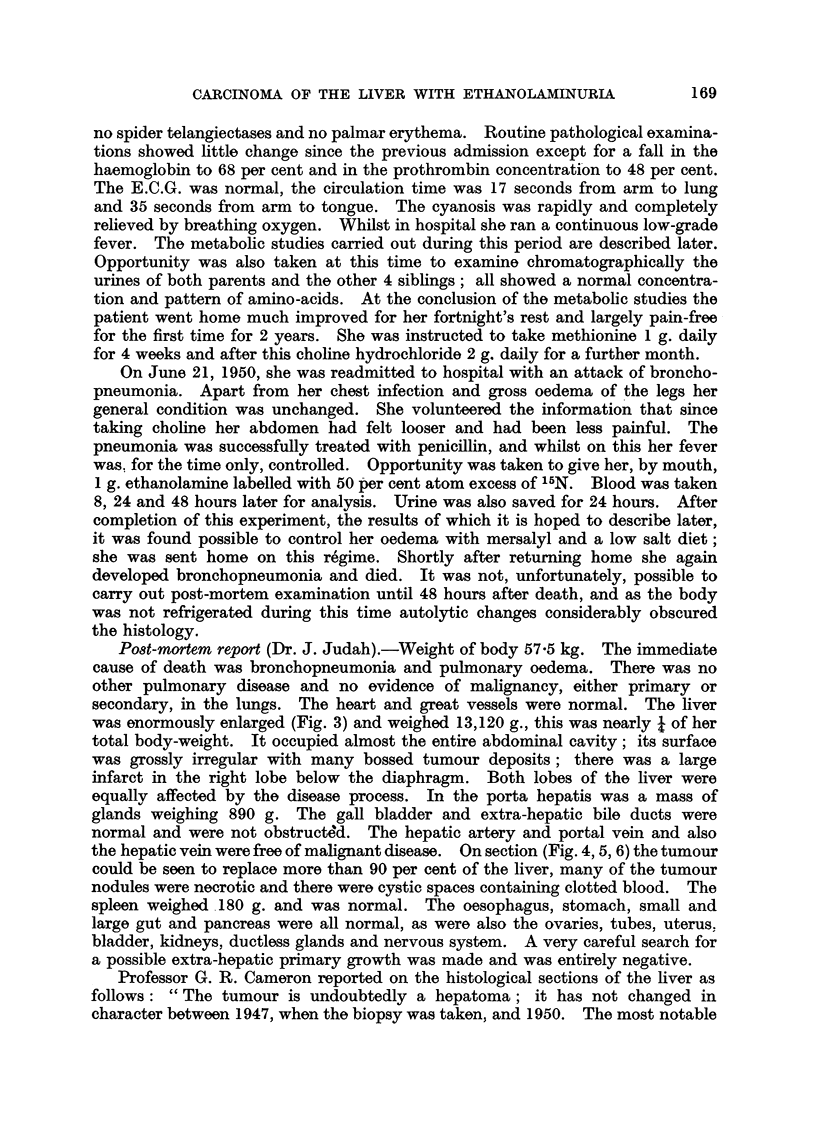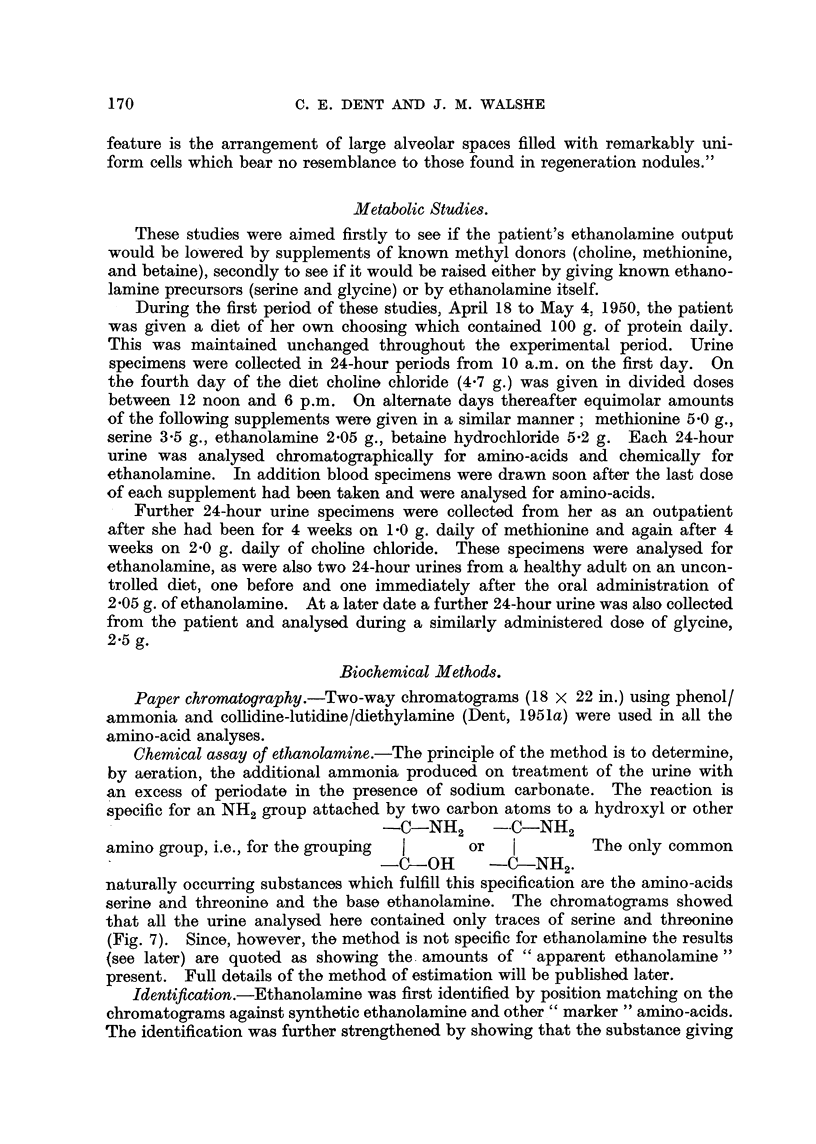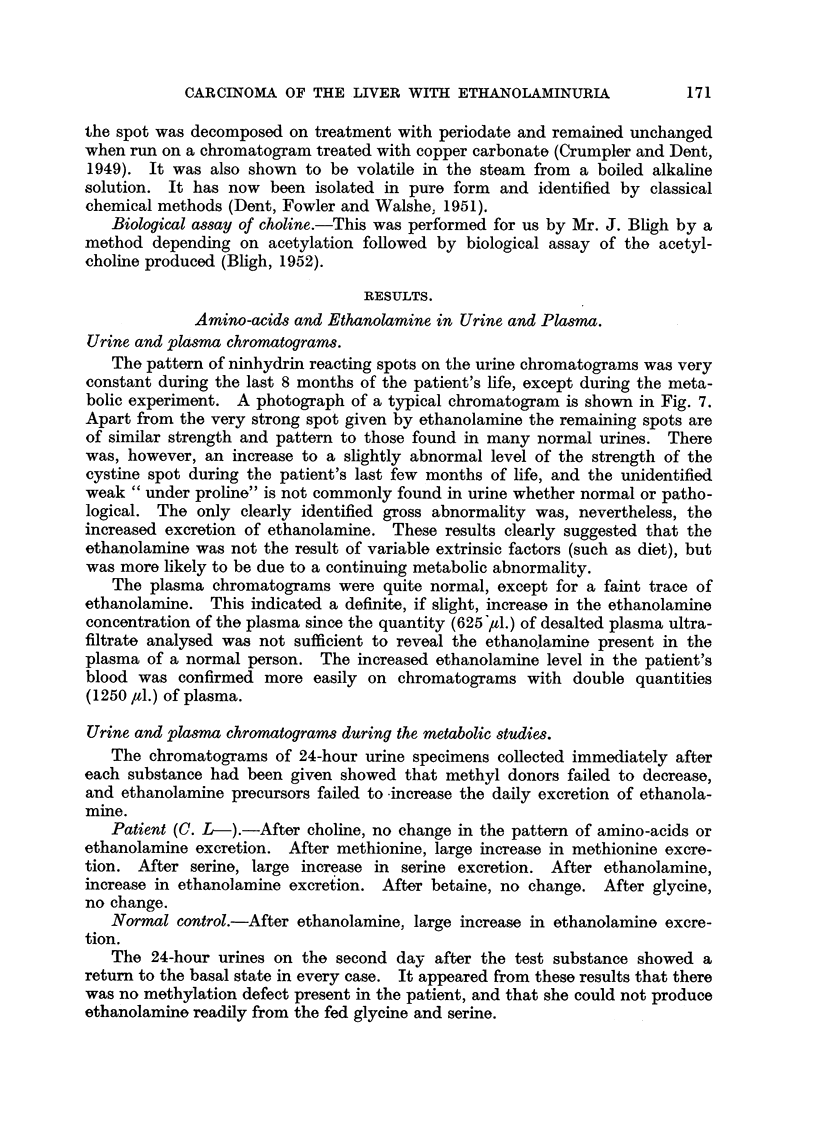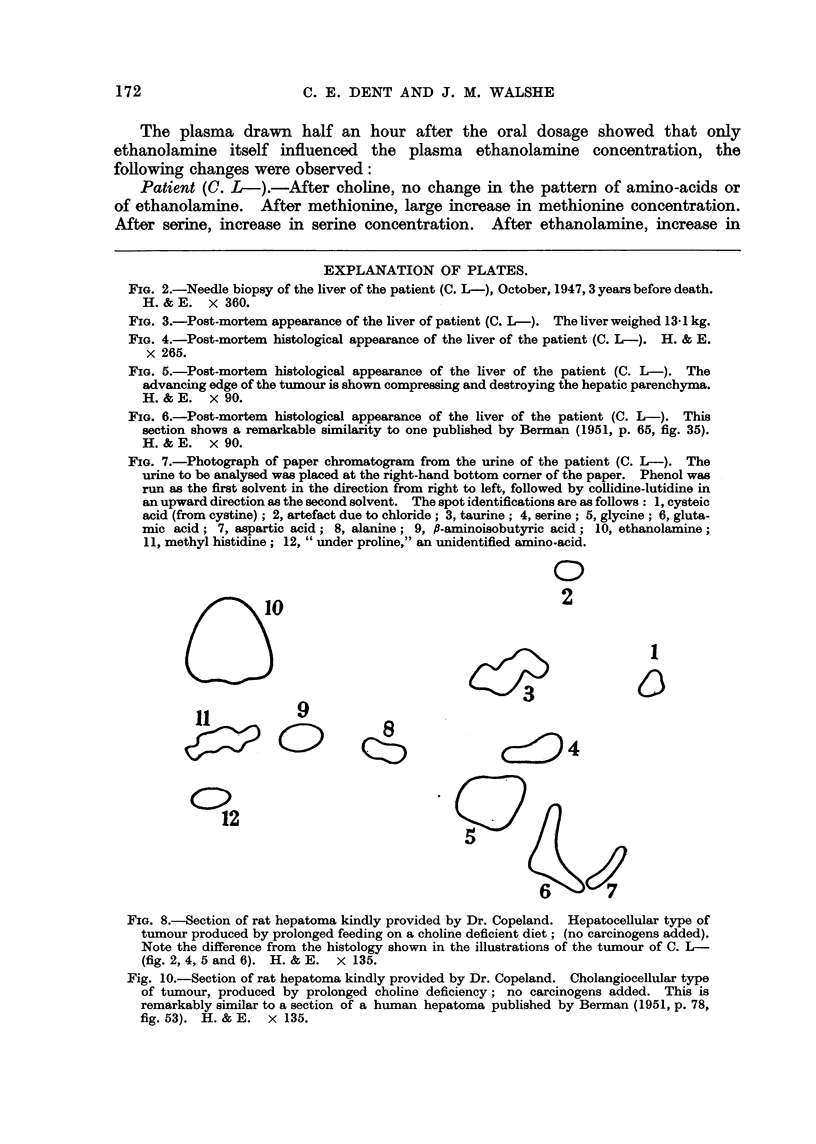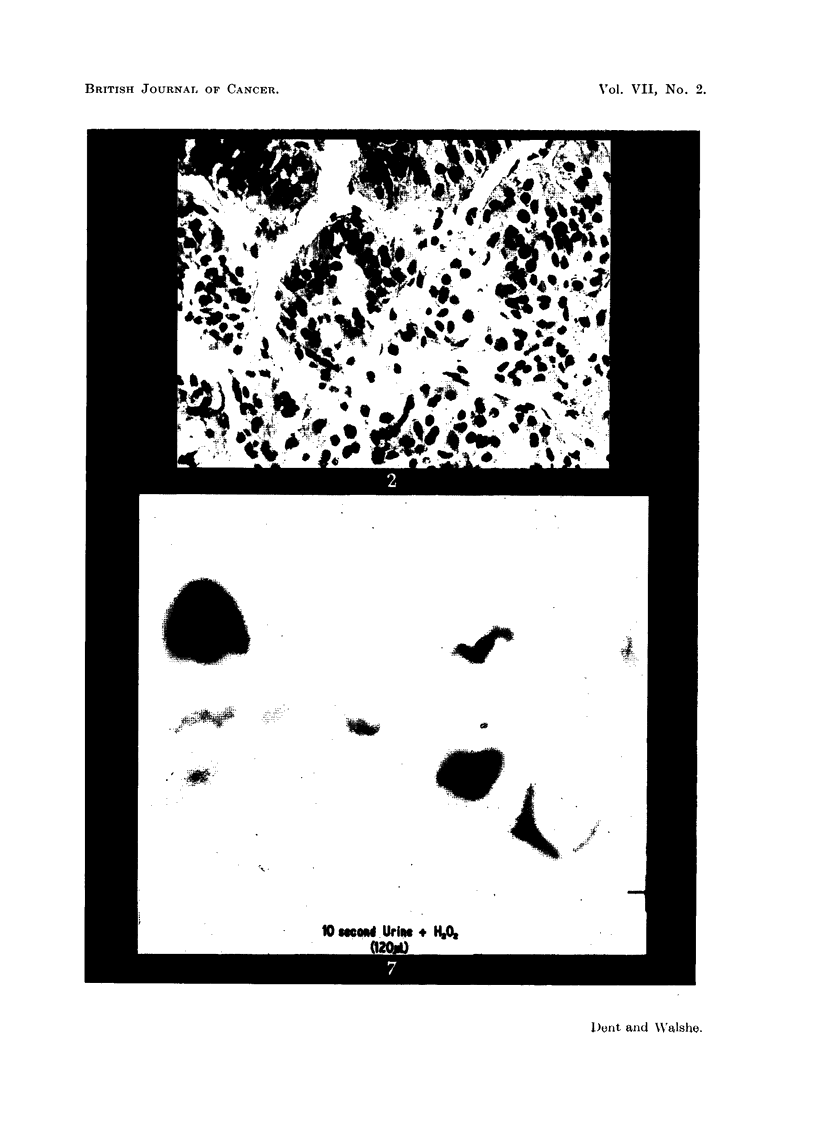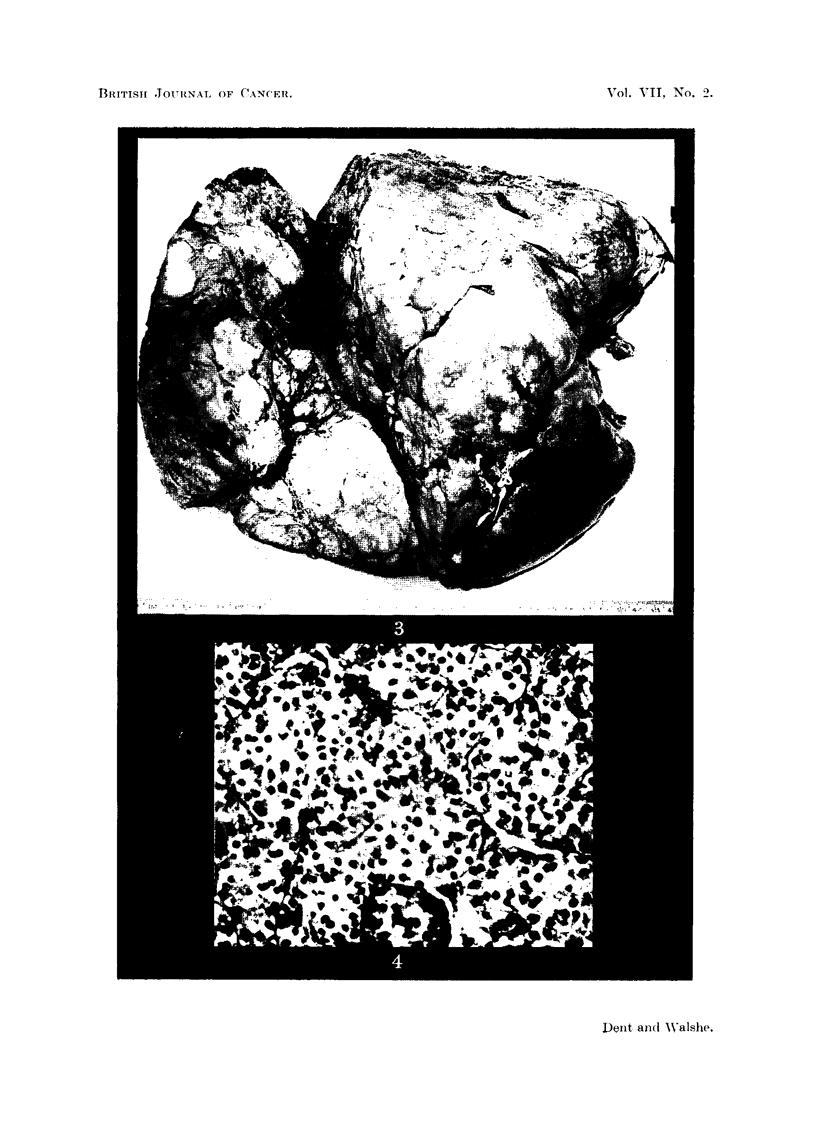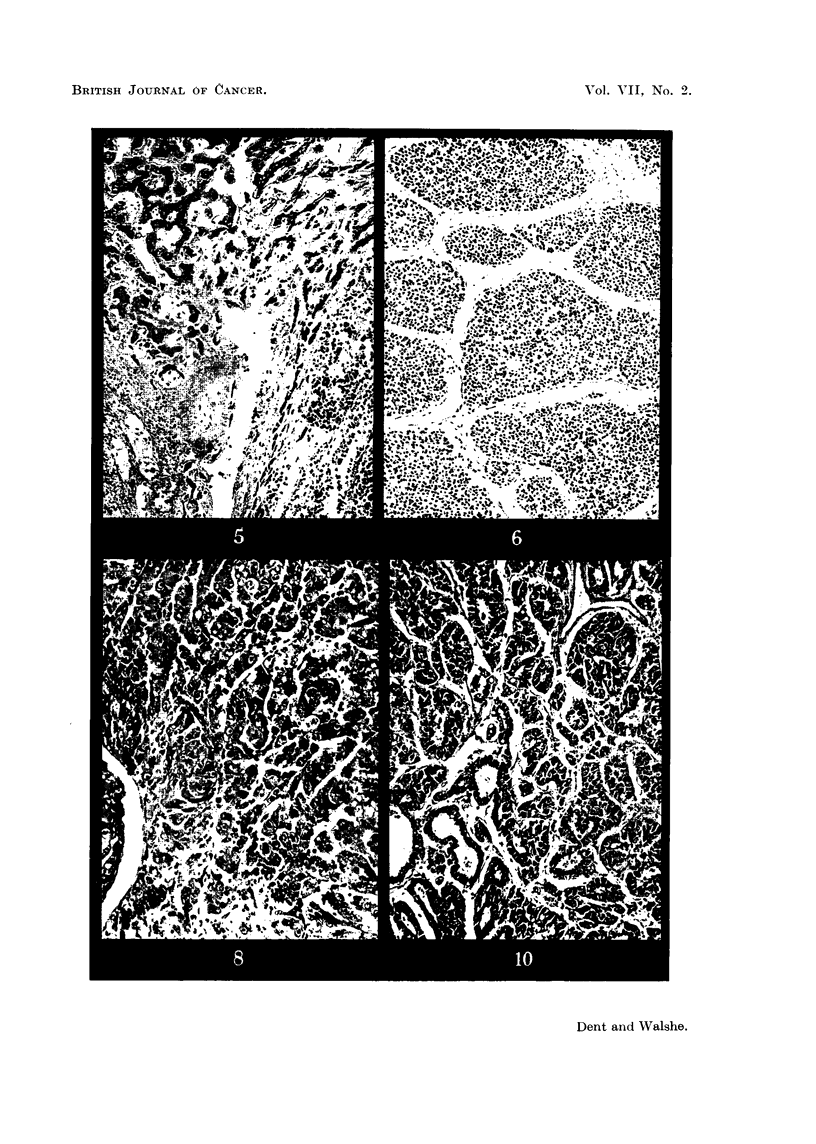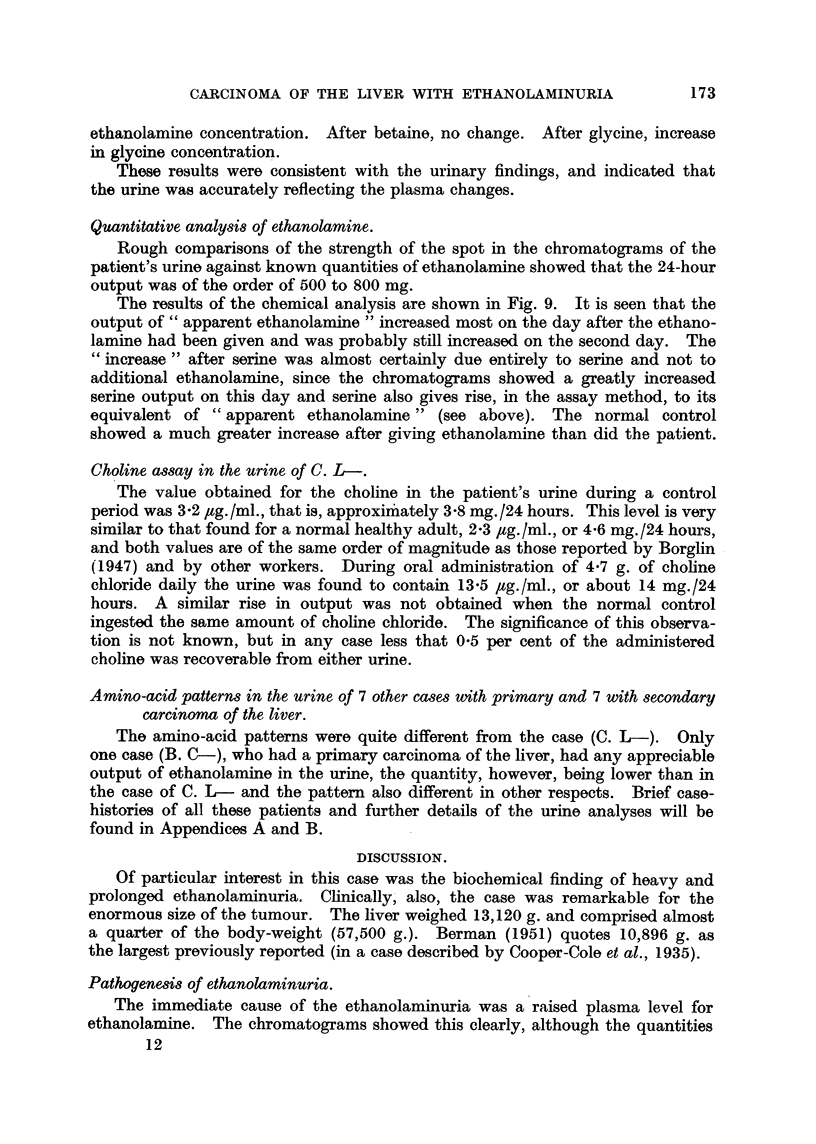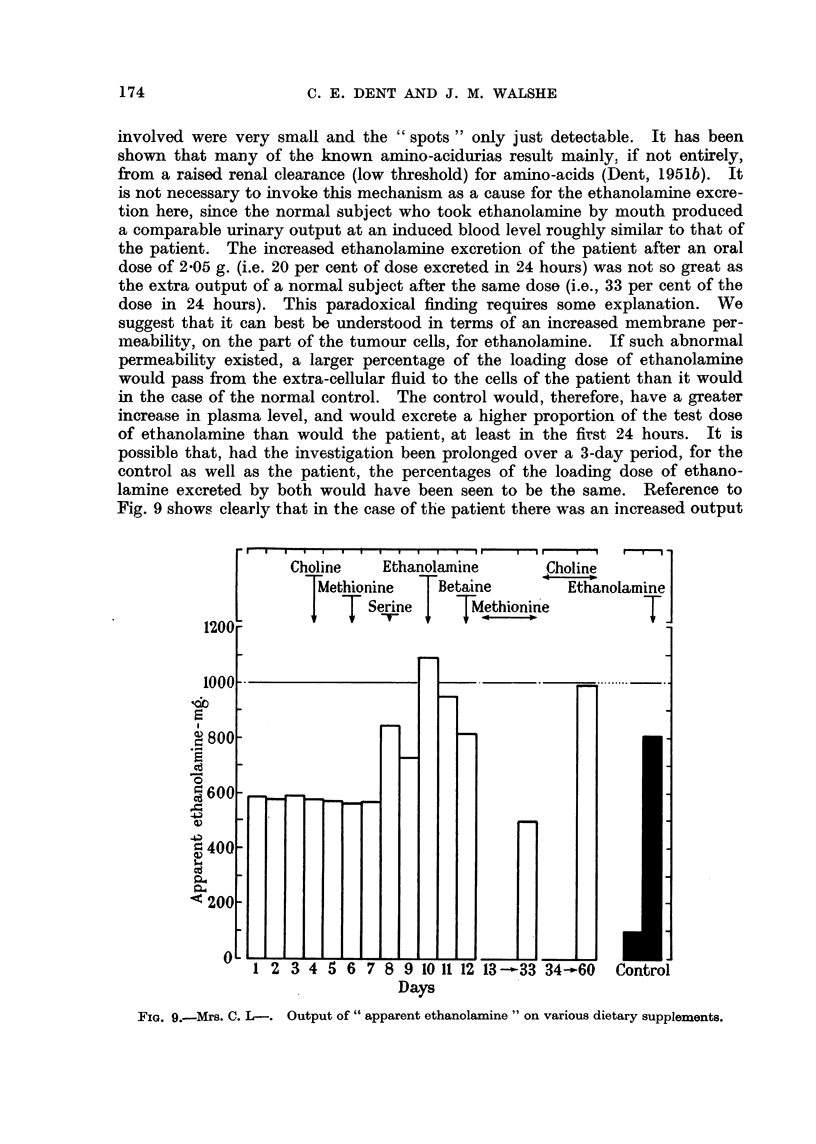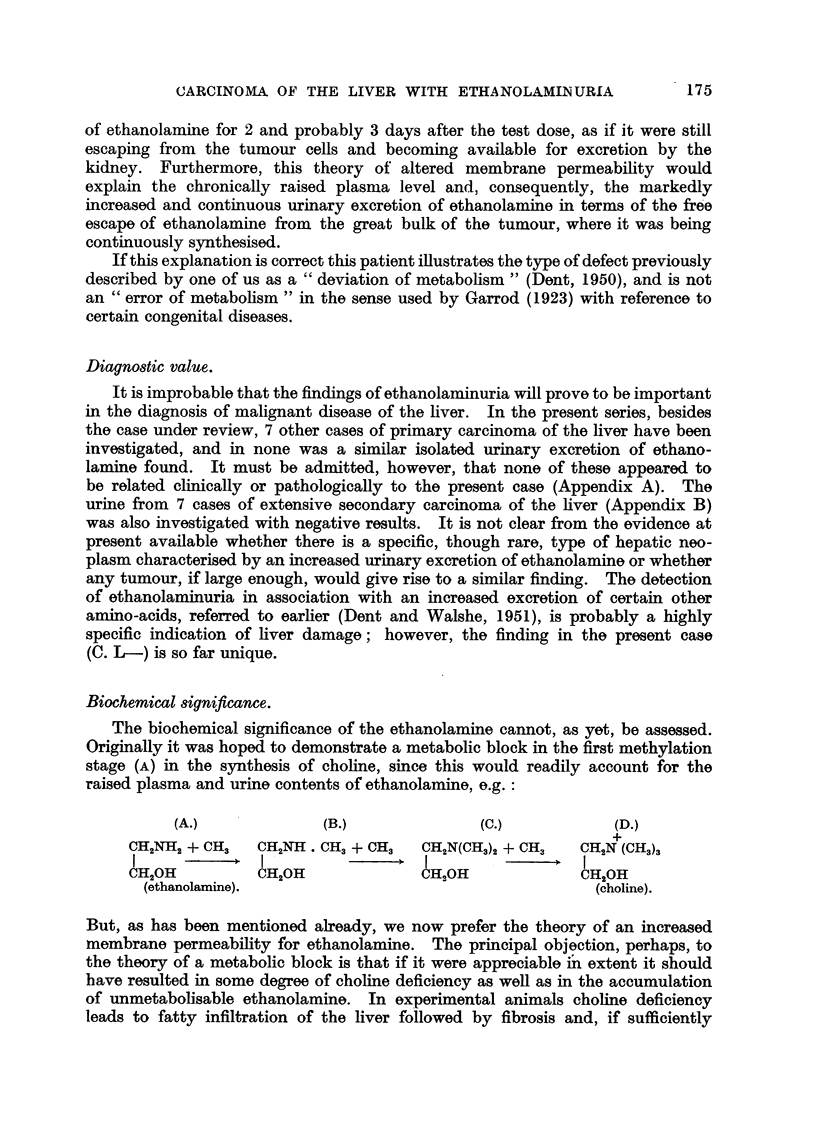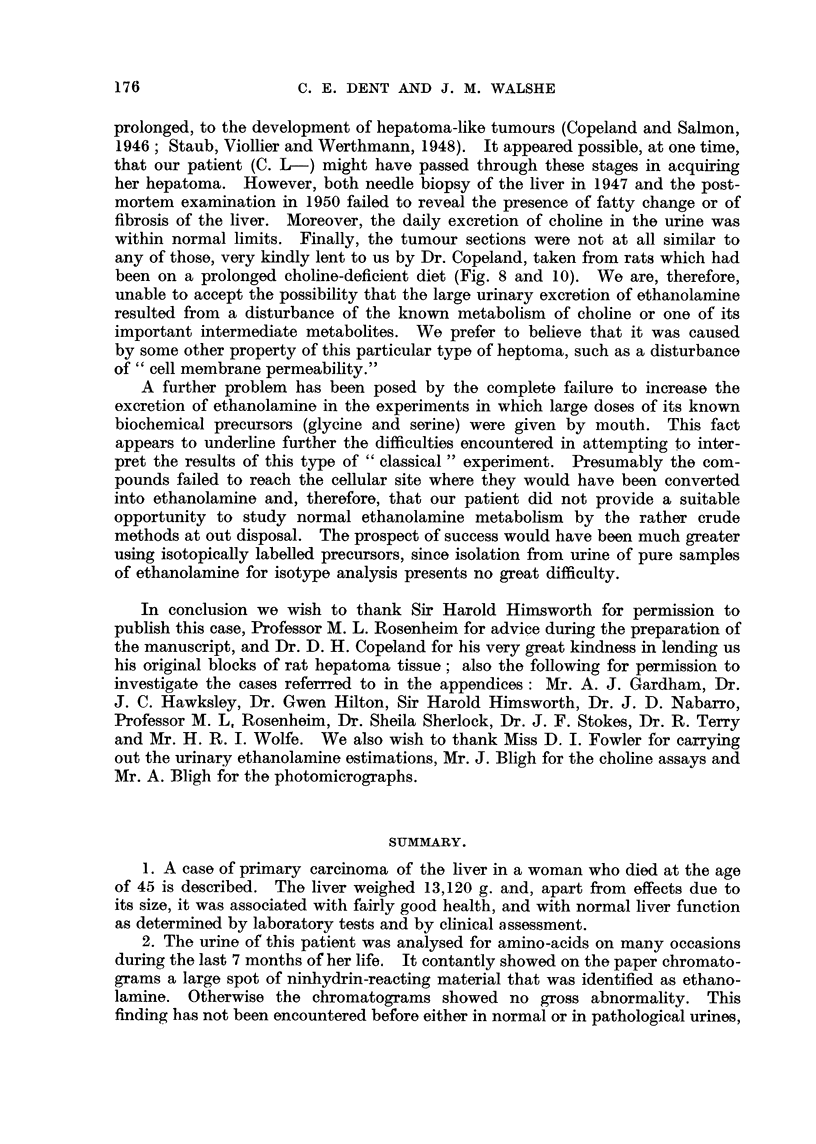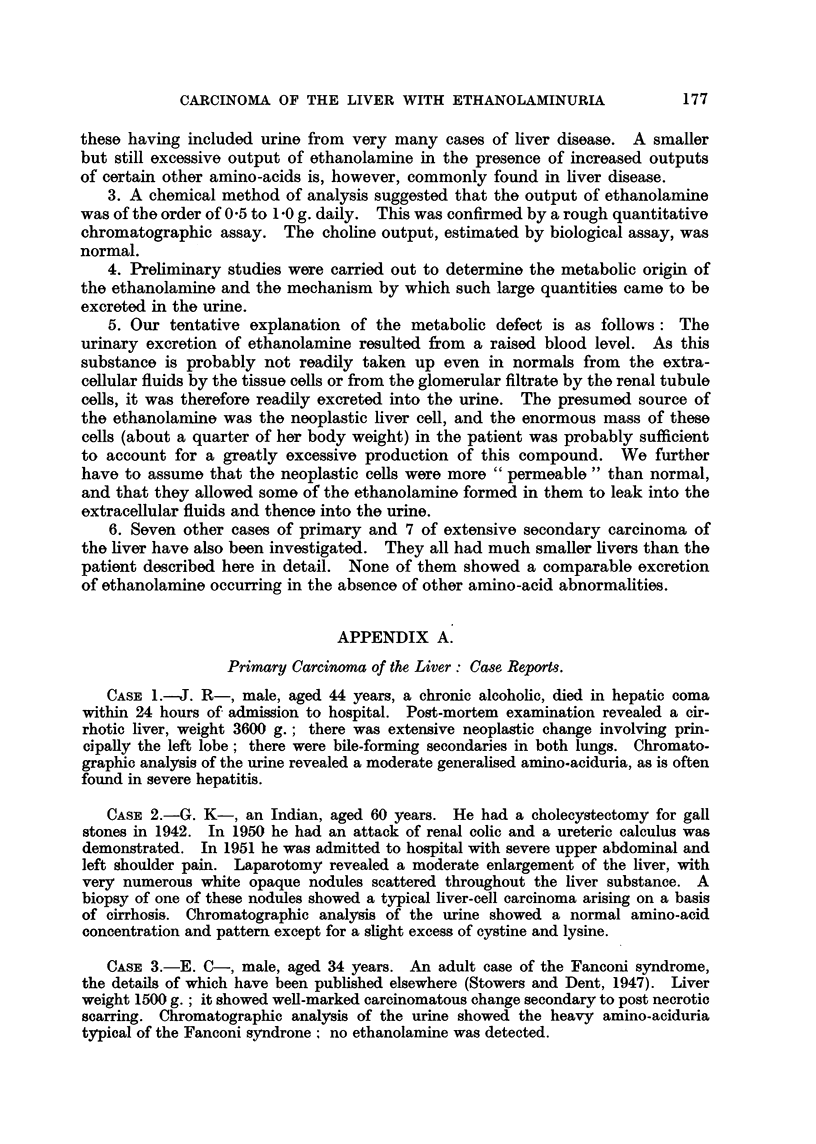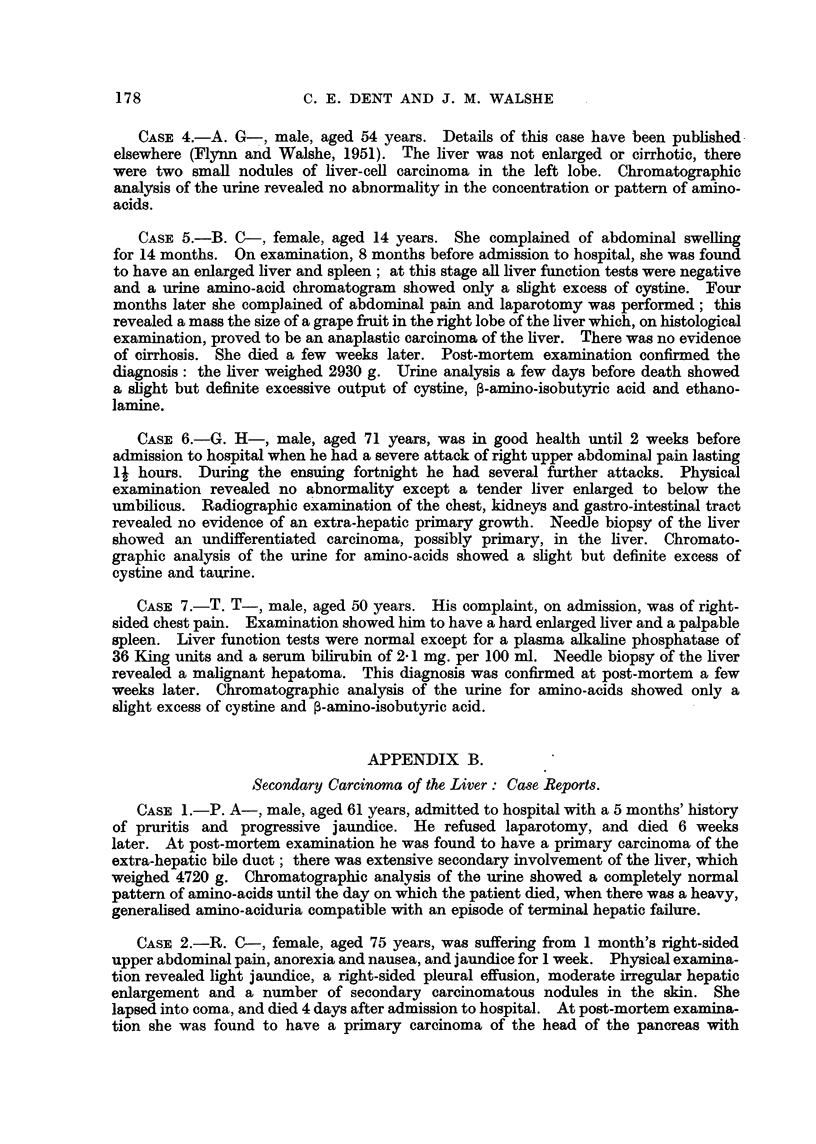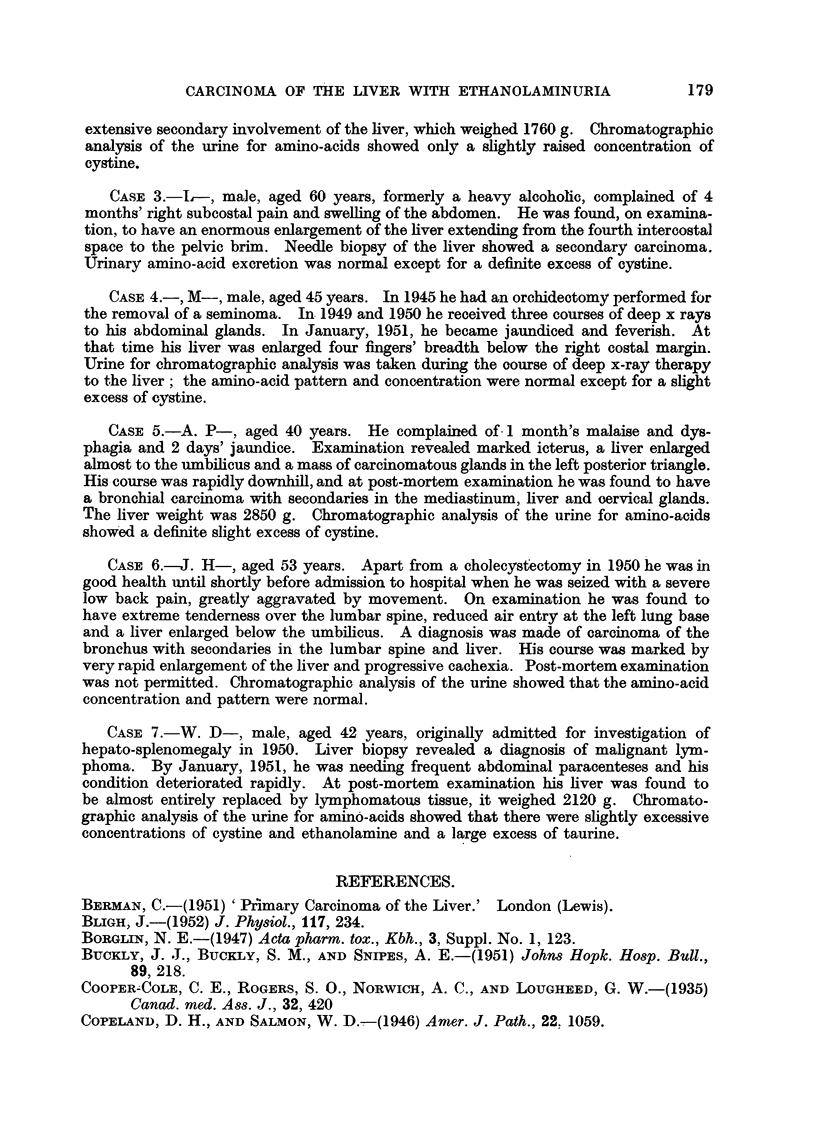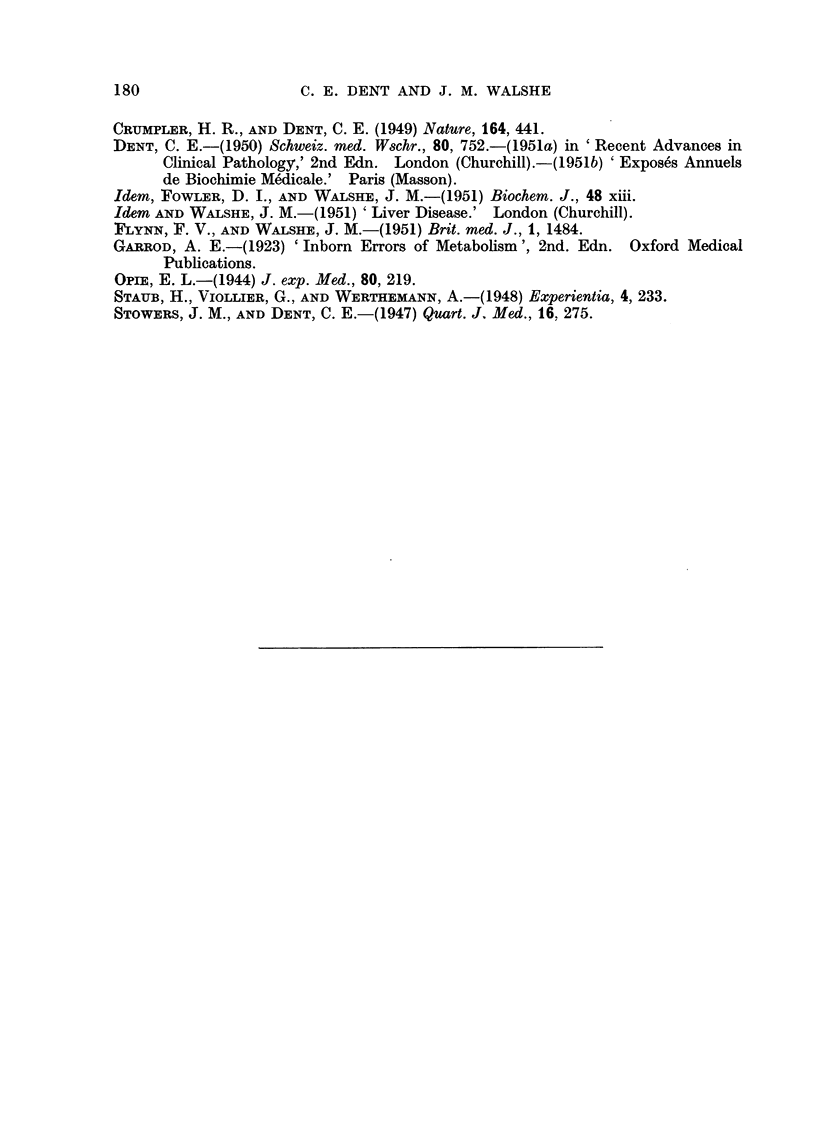# Primary Carcinoma of the Liver: Description of a Case with Ethanolaminuria, A New and Obscure Metabolic Defect

**DOI:** 10.1038/bjc.1953.16

**Published:** 1953-06

**Authors:** C. E. Dent, J. M. Walshe

## Abstract

**Images:**


### PRIMARY CARCINOMA OF THE LIVER: DESCRIPTION OF A CASE WITH ETHANOLAMINURIA, A NEW AND OBSCURE METABOLIC DEFECT.

C. E. DENT AND J. M. WALSHE.

*From the Medical Unit, University College Hospital.*

Received for publication February 27, 1953.

In the course of a study of amino-acid metabolism in liver disease grossly abnormal amounts of ethanolamine (amino-ethanol, $CH_2(NH_2) . CH_2OH$) were found in the urine of a patient with primary carcinoma of the liver (Dent and Walshe, 1951). No other amino compounds were found in abnormal amounts in the urine, and the excretion of ethanolamine persisted unaltered throughout the remaining 7 months of life. The same study indicated that an excessive output of ethanolamine frequently occurred in the urine of patients with severe parenchymal liver disease, but that in such cases it was always accompanied by an excess of many other amino-acids. The presence in the urine of large quantities of ethanolamine, as an isolated abnormality, in the patient with primary carcinoma of the liver was therefore of some interest. Its excretion might have resulted from a disorder of metabolism consequent on non-specific liver damage or from some peculiar form of neoplastic cell metabolism; or it might even have represented a hitherto unrecognised error of metabolism which preceded and led to the development of a primary carcinoma in the liver.

It has for some years been recognised, as a result mainly of animal experiment, that ethanolamine plays an important role as an intermediary in the metabolism of phospholipids and amino-acids. The metabolic inter-relationship between these compounds is shown in the accompanying figure (Fig. 1).

Fig. 1.—Some metabolic inter-relationships involving ethanolamine and other compounds.

Experimental dietary deficiency of choline has also been extensively studied. In rats it has been shown to lead to the production of fatty livers, followed after some time by cirrhosis. More recent work has shown that this may be followed eventually by the development of hepatoma-like tumours (Copeland and Salmon, 1946; Staub, Viollier and Werthemann, 1948). Furthermore, choline deficiency of a severity not sufficient to produce cirrhosis predisposes the liver to the carcinogenic action of Butter Yellow (Opie, 1944; Buckley, Buckley and Snipes, 1951). In the human hepatomata that occur in tropical countries there is ample evidence that dietary factors are concerned (Berman, 1951). The possibility that the continued loss of ethanolamine in the urine of our patient might have been the primary metabolic defect leading to a conditioned choline deficiency and eventually to hepatoma formation had therefore to be considered seriously. With this possibility in mind, it may be noted that certain clinical features were unusual; the patient survived in comparatively good health for over 3 years after the original discovery of hepatic enlargement, at post-mortem examination no fibrosis was found in the small amount of remaining liver tissue and, apart from the adjacent lymph glands in the porta hepatis, no secondary tumours were found.

Once the ethanolaminuria in our patient was detected and confirmed unambiguously by chemical means (Dent, Fowler and Walshe, 1951), the opportunity was taken to carry out a series of metabolic studies on the patient. Further, since the presence of ethanolamine in the urine might have proved to be a genetically linked error of metabolism, or of diagnostic value, investigations were also carried out on urine from the patient's parents and siblings and on a series of cases of primary and secondary carcinoma of the liver. The results of these later studies are here reported.

*Case history.*

Mrs. C. L—, a housewife, was aged 45 years at the time of her death in 1950. Apart from an attack of jaundice at the age of 7 she was in good health until April, 1939, when, at the age of 34, she had a febrile illness with delirium and a productive cough. She had a second similar attack in October that year, and this, like the first, lasted about 3 weeks. She suffered from similar attacks each winter until 1942. In that year her illness was more severe and was associated with generalised aches and pains and vomiting. Since that time she suffered from morning vomiting and also a burning pain, relieved by alkalis, behind the lower end of her sternum. Despite these regular winter illnesses and an increasing sense of tiredness she continued with her work in a munitions factory until 1945. From then until her death in 1950 she worked as a housewife.

In January, 1947, she first noticed a painless lump in her abdomen. In March of that year, following an illness with fever and malaise, she noticed that her abdomen was becoming tender to touch, her appetite was failing and the morning vomiting was becoming more severe. It was at this time that she was first seen and admitted for investigation at University College Hospital. Both parents and her 4 siblings were all alive and well. Examination revealed a pale, slightly cyanosed, nonicteric woman of 8 st. 10 lb. (55·5 kg.). The cardiovascular system was normal and the blood pressure 115/80 mm. Hg. There were numerous rhonchi scattered over both lung fields. The central nervous system was normal. The abdomen was distended by a considerable enlargement of the liver, which had a firm irregular edge stretching from the left hypochondrium almost to the right iliac fossa. Neither the kidneys nor the spleen could be felt. Details of some of the pathological examinations made at that time are given in the accompanying table (Table I). In addition, the peripheral blood and sternal marrow were normal, the sedimentation rate was raised, the prothrombin concentration and serum flocculation tests were normal, there was no occult blood in the stools, the Wassermann reaction was negative and free hydrochloric acid was present in the stomach. Straight X-ray examination of the chest and abdomen revealed no abnormality. It was concluded that she had secondary carcinoma of the liver, the primary growth not having been found.

### TABLE I.

| Date. | Hippuric acid synthesis. | Prothrombin concentration (% normal). | Serum albumin (g. per 100 ml.). | Serum globulin (g. per 100 ml.). | Serum flocculation tests.* | Serum bilirubin (mg. per 100 ml.). | Plasma cholesterol. (mg. per 100 ml.) | Alkaline phosphatase (King units per 100 ml.). | Bromsulphalein clearance (2 mg./kg./ 30 min.). | Red cell cholinesterase ($Q/CO_2$/ ml./hr.).† | Plasma cholinesterase ($Q/CO_2$/ ml./hr.).† | Fasting blood sugar (mg. 100 ml.). |
|---|---|---|---|---|---|---|---|---|---|---|---|---|
| 8.iv.47 | 2·83 g or 94% | — | 4·1 | 2·4 | –ve | — | — | — | — | — | — | — |
| 24.x.47 | 127% | 120 | — | — | –ve | — | — | — | — | — | — | — |
| 4.xii.48 | — | — | — | — | — | — | — | — | — | 1767 | 1215 | — |
| 27.iv.49 | — | — | 4·0 | 3·2 | — | — | — | — | — | 1841 | 1346 | — |
| 29.viii.49 | — | — | 4·1 | 3·3 | — | — | — | — | — | 1606 | 1199 | — |
| 24.i.50 | — | — | 4·2 | 3·3 | –ve | less than 0·4 | — | — | 5% retained | 1206 | 1249 | — |
| 18.iv.50 | — | 71 | — | — | –ve | 0·4 | — | — | — | — | — | 125 |
| 2.v.50 | — | 48 | — | — | — | — | — | — | — | — | — | — |
| 27.v.50 | — | — | — | — | — | — | — | 44 | — | — | — | — |
| 22.vi.50 | — | 67 | 4·1 | 2·1 | –ve | 0·7 | 200 | — | — | — | — | — |

* Serum flocculation tests = colloidal red, cephalin cholesterol, thymol turbidity and flocculation.
† Lower limits of normal = 1000 units.

27.v.50 Electrophoretic pattern of plasma proteins (Tiselius) by Dr. N. Martin. Albumin 45%, $\alpha$-globulin 14%, $\beta$-globulin 24%, $\gamma$-globulin 17%.

In October, 1947, because of her continued good general condition, she was readmitted for further investigation ; all tests performed in March were repeated and were again normal ; in addition a barium meal examination was performed and this was also normal. Needle biopsy of the liver revealed a hepatoma (Fig. 2) of the liver-cell type without any evidence of fatty infiltration or fibrosis.

During 1948 she complained of occasional back pain and a gripping pain in her abdomen, mostly in the left flank and along the lower border of the liver. She was able to carry on with her housework, her appetite was still good and her bowels regular ; she was, however, troubled by occasional nose bleeds. During the year her abdominal girth increased from 89 to 94 cm. In 1949 there was little change in her general condition except for the development of ankle oedema ; the liver continued to enlarge and she had rather more abdominal pain. Her weight remained steady and the biochemical tests of liver function were all normal. It was in December of this year that a urinary chromatogram was first carried out, and this revealed a large excess of a ninhydrin reactor later identified as ethanolamine by specific chromatographic techniques and by isolation in the pure state (Dent, Fowler and Walshe, 1951).

During the first 4 months of 1950 abdominal pain became more severe and the liver continued to enlarge. Repeated examinations of the urine continued to reveal the presence of ethanolamine as a persistant abnormality and in April she was admitted for metabolic studies. Examination revealed a small wasted woman, weight 8 stone 10 lb., with marked cyanosis, clubbing of the fingers, capillary pulsation, intense engorgement of the superficial veins over the chest and abdomen and gross abdominal distension. In the heart there was a loud rough systolic murmur all over the precordium, maximal at the apex. The pulse was regular at 90 and the blood-pressure was 110/60 mm. Hg. There were rhonchi over both lung fields and bilateral basal rales. In the nervous system the pupils were normal, the knee- and ankle-jerks were absent and the plantar responses were flexor. The abdominal girth had increased to 109 cm., the liver edge was almost at the pelvic brim and extended into both flanks ; the spleen was not palpable, nor was it possible to demonstrate free fluid. Pelvic examination was normal. There was moderate oedema localized to the feet. There were no spider telangiectases and no palmar erythema. Routine pathological examinations showed little change since the previous admission except for a fall in the haemoglobin to 68 per cent and in the prothrombin concentration to 48 per cent. The E.C.G. was normal, the circulation time was 17 seconds from arm to lung and 35 seconds from arm to tongue. The cyanosis was rapidly and completely relieved by breathing oxygen. Whilst in hospital she ran a continuous low-grade fever. The metabolic studies carried out during this period are described later. Opportunity was also taken at this time to examine chromatographically the urines of both parents and the other 4 siblings ; all showed a normal concentration and pattern of amino-acids. At the conclusion of the metabolic studies the patient went home much improved for her fortnight's rest and largely pain-free for the first time for 2 years. She was instructed to take methionine 1 g. daily for 4 weeks and after this choline hydrochloride 2 g. daily for a further month.

On June 21, 1950, she was readmitted to hospital with an attack of bronchopneumonia. Apart from her chest infection and gross oedema of the legs her general condition was unchanged. She volunteered the information that since taking choline her abdomen had felt looser and had been less painful. The pneumonia was successfully treated with penicillin, and whilst on this her fever was, for the time only, controlled. Opportunity was taken to give her, by mouth, 1 g. ethanolamine labelled with 50 per cent atom excess of $^{15}N$. Blood was taken 8, 24 and 48 hours later for analysis. Urine was also saved for 24 hours. After completion of this experiment, the results of which it is hoped to describe later, it was found possible to control her oedema with mersalyl and a low salt diet ; she was sent home on this régime. Shortly after returning home she again developed bronchopneumonia and died. It was not, unfortunately, possible to carry out post-mortem examination until 48 hours after death, and as the body was not refrigerated during this time autolytic changes considerably obscured the histology.

*Post-mortem report* (Dr. J. Judah).—Weight of body 57·5 kg. The immediate cause of death was bronchopneumonia and pulmonary oedema. There was no other pulmonary disease and no evidence of malignancy, either primary or secondary, in the lungs. The heart and great vessels were normal. The liver was enormously enlarged (Fig. 3) and weighed 13,120 g., this was nearly $\frac{1}{4}$ of her total body-weight. It occupied almost the entire abdominal cavity ; its surface was grossly irregular with many bossed tumour deposits ; there was a large infarct in the right lobe below the diaphragm. Both lobes of the liver were equally affected by the disease process. In the porta hepatis was a mass of glands weighing 890 g. The gall bladder and extra-hepatic bile ducts were normal and were not obstructed. The hepatic artery and portal vein and also the hepatic vein were free of malignant disease. On section (Fig. 4, 5, 6) the tumour could be seen to replace more than 90 per cent of the liver, many of the tumour nodules were necrotic and there were cystic spaces containing clotted blood. The spleen weighed 180 g. and was normal. The oesophagus, stomach, small and large gut and pancreas were all normal, as were also the ovaries, tubes, uterus, bladder, kidneys, ductless glands and nervous system. A very careful search for a possible extra-hepatic primary growth was made and was entirely negative.

Professor G. R. Cameron reported on the histological sections of the liver as follows : " The tumour is undoubtedly a hepatoma ; it has not changed in character between 1947, when the biopsy was taken, and 1950. The most notable feature is the arrangement of large alveolar spaces filled with remarkably uniform cells which bear no resemblance to those found in regeneration nodules."

### *Metabolic Studies.*

These studies were aimed firstly to see if the patient's ethanolamine output would be lowered by supplements of known methyl donors (choline, methionine, and betaine), secondly to see if it would be raised either by giving known ethanolamine precursors (serine and glycine) or by ethanolamine itself.

During the first period of these studies, April 18 to May 4, 1950, the patient was given a diet of her own choosing which contained 100 g. of protein daily. This was maintained unchanged throughout the experimental period. Urine specimens were collected in 24-hour periods from 10 a.m. on the first day. On the fourth day of the diet choline chloride (4·7 g.) was given in divided doses between 12 noon and 6 p.m. On alternate days thereafter equimolar amounts of the following supplements were given in a similar manner; methionine 5·0 g., serine 3·5 g., ethanolamine 2·05 g., betaine hydrochloride 5·2 g. Each 24-hour urine was analysed chromatographically for amino-acids and chemically for ethanolamine. In addition blood specimens were drawn soon after the last dose of each supplement had been taken and were analysed for amino-acids.

Further 24-hour urine specimens were collected from her as an outpatient after she had been for 4 weeks on 1·0 g. daily of methionine and again after 4 weeks on 2·0 g. daily of choline chloride. These specimens were analysed for ethanolamine, as were also two 24-hour urines from a healthy adult on an uncontrolled diet, one before and one immediately after the oral administration of 2·05 g. of ethanolamine. At a later date a further 24-hour urine was also collected from the patient and analysed during a similarly administered dose of glycine, 2·5 g.

### *Biochemical Methods.*

*Paper chromatography.*—Two-way chromatograms (18 × 22 in.) using phenol/ammonia and collidine-lutidine/diethylamine (Dent, 1951*a*) were used in all the amino-acid analyses.

*Chemical assay of ethanolamine.*—The principle of the method is to determine, by aeration, the additional ammonia produced on treatment of the urine with an excess of periodate in the presence of sodium carbonate. The reaction is specific for an $NH_2$ group attached by two carbon atoms to a hydroxyl or other amino group, i.e., for the grouping

$$\begin{array}{c}-C-NH_2\\ |\\ -C-OH\end{array} \quad \text{or} \quad \begin{array}{c}-C-NH_2\\ |\\ -C-NH_2.\end{array}$$

The only common naturally occurring substances which fulfill this specification are the amino-acids serine and threonine and the base ethanolamine. The chromatograms showed that all the urine analysed here contained only traces of serine and threonine (Fig. 7). Since, however, the method is not specific for ethanolamine the results (see later) are quoted as showing the amounts of "apparent ethanolamine" present. Full details of the method of estimation will be published later.

*Identification.*—Ethanolamine was first identified by position matching on the chromatograms against synthetic ethanolamine and other "marker" amino-acids. The identification was further strengthened by showing that the substance giving the spot was decomposed on treatment with periodate and remained unchanged when run on a chromatogram treated with copper carbonate (Crumpler and Dent, 1949). It was also shown to be volatile in the steam from a boiled alkaline solution. It has now been isolated in pure form and identified by classical chemical methods (Dent, Fowler and Walshe, 1951).

*Biological assay of choline.*—This was performed for us by Mr. J. Bligh by a method depending on acetylation followed by biological assay of the acetylcholine produced (Bligh, 1952).

### RESULTS.

### *Amino-acids and Ethanolamine in Urine and Plasma.*

*Urine and plasma chromatograms.*

The pattern of ninhydrin reacting spots on the urine chromatograms was very constant during the last 8 months of the patient's life, except during the metabolic experiment. A photograph of a typical chromatogram is shown in Fig. 7. Apart from the very strong spot given by ethanolamine the remaining spots are of similar strength and pattern to those found in many normal urines. There was, however, an increase to a slightly abnormal level of the strength of the cystine spot during the patient's last few months of life, and the unidentified weak " under proline" is not commonly found in urine whether normal or pathological. The only clearly identified gross abnormality was, nevertheless, the increased excretion of ethanolamine. These results clearly suggested that the ethanolamine was not the result of variable extrinsic factors (such as diet), but was more likely to be due to a continuing metabolic abnormality.

The plasma chromatograms were quite normal, except for a faint trace of ethanolamine. This indicated a definite, if slight, increase in the ethanolamine concentration of the plasma since the quantity (625 $\mu$l.) of desalted plasma ultrafiltrate analysed was not sufficient to reveal the ethanolamine present in the plasma of a normal person. The increased ethanolamine level in the patient's blood was confirmed more easily on chromatograms with double quantities (1250 $\mu$l.) of plasma.

*Urine and plasma chromatograms during the metabolic studies.*

The chromatograms of 24-hour urine specimens collected immediately after each substance had been given showed that methyl donors failed to decrease, and ethanolamine precursors failed to increase the daily excretion of ethanolamine.

*Patient* (*C. L*—).—After choline, no change in the pattern of amino-acids or ethanolamine excretion. After methionine, large increase in methionine excretion. After serine, large increase in serine excretion. After ethanolamine, increase in ethanolamine excretion. After betaine, no change. After glycine, no change.

*Normal control.*—After ethanolamine, large increase in ethanolamine excretion.

The 24-hour urines on the second day after the test substance showed a return to the basal state in every case. It appeared from these results that there was no methylation defect present in the patient, and that she could not produce ethanolamine readily from the fed glycine and serine.

The plasma drawn half an hour after the oral dosage showed that only ethanolamine itself influenced the plasma ethanolamine concentration, the following changes were observed:

*Patient* (*C. L*—).—After choline, no change in the pattern of amino-acids or of ethanolamine. After methionine, large increase in methionine concentration. After serine, increase in serine concentration. After ethanolamine, increase in

---

EXPLANATION OF PLATES.

FIG. 2.—Needle biopsy of the liver of the patient (C. L—), October, 1947, 3 years before death. H. & E. × 360.

FIG. 3.—Post-mortem appearance of the liver of patient (C. L—). The liver weighed 13·1 kg.

FIG. 4.—Post-mortem histological appearance of the liver of the patient (C. L—). H. & E. × 265.

FIG. 5.—Post-mortem histological appearance of the liver of the patient (C. L—). The advancing edge of the tumour is shown compressing and destroying the hepatic parenchyma. H. & E. × 90.

FIG. 6.—Post-mortem histological appearance of the liver of the patient (C. L—). This section shows a remarkable similarity to one published by Berman (1951, p. 65, fig. 35). H. & E. × 90.

FIG. 7.—Photograph of paper chromatogram from the urine of the patient (C. L—). The urine to be analysed was placed at the right-hand bottom corner of the paper. Phenol was run as the first solvent in the direction from right to left, followed by collidine-lutidine in an upward direction as the second solvent. The spot identifications are as follows : 1, cysteic acid (from cystine) ; 2, artefact due to chloride ; 3, taurine ; 4, serine ; 5, glycine ; 6, glutamic acid ; 7, aspartic acid ; 8, alanine ; 9, β-aminoisobutyric acid ; 10, ethanolamine ; 11, methyl histidine ; 12, " under proline," an unidentified amino-acid.

FIG. 8.—Section of rat hepatoma kindly provided by Dr. Copeland. Hepatocellular type of tumour produced by prolonged feeding on a choline deficient diet ; (no carcinogens added). Note the difference from the histology shown in the illustrations of the tumour of C. L— (fig. 2, 4, 5 and 6). H. & E. × 135.

Fig. 10.—Section of rat hepatoma kindly provided by Dr. Copeland. Cholangiocellular type of tumour, produced by prolonged choline deficiency ; no carcinogens added. This is remarkably similar to a section of a human hepatoma published by Berman (1951, p. 78, fig. 53). H. & E. × 135.

2

7

Dent and Walshe.

3

4

5

6

8

10

Dent and Walshe.

ethanolamine concentration. After betaine, no change. After glycine, increase in glycine concentration.

These results were consistent with the urinary findings, and indicated that the urine was accurately reflecting the plasma changes.

*Quantitative analysis of ethanolamine.*

Rough comparisons of the strength of the spot in the chromatograms of the patient's urine against known quantities of ethanolamine showed that the 24-hour output was of the order of 500 to 800 mg.

The results of the chemical analysis are shown in Fig. 9. It is seen that the output of " apparent ethanolamine " increased most on the day after the ethanolamine had been given and was probably still increased on the second day. The " increase " after serine was almost certainly due entirely to serine and not to additional ethanolamine, since the chromatograms showed a greatly increased serine output on this day and serine also gives rise, in the assay method, to its equivalent of " apparent ethanolamine " (see above). The normal control showed a much greater increase after giving ethanolamine than did the patient.

*Choline assay in the urine of C. L—.*

The value obtained for the choline in the patient's urine during a control period was 3·2 $\mu$g./ml., that is, approximately 3·8 mg./24 hours. This level is very similar to that found for a normal healthy adult, 2·3 $\mu$g./ml., or 4·6 mg./24 hours, and both values are of the same order of magnitude as those reported by Borglin (1947) and by other workers. During oral administration of 4·7 g. of choline chloride daily the urine was found to contain 13·5 $\mu$g./ml., or about 14 mg./24 hours. A similar rise in output was not obtained when the normal control ingested the same amount of choline chloride. The significance of this observation is not known, but in any case less that 0·5 per cent of the administered choline was recoverable from either urine.

*Amino-acid patterns in the urine of* 7 *other cases with primary and* 7 *with secondary carcinoma of the liver.*

The amino-acid patterns were quite different from the case (C. L—). Only one case (B. C—), who had a primary carcinoma of the liver, had any appreciable output of ethanolamine in the urine, the quantity, however, being lower than in the case of C. L— and the pattern also different in other respects. Brief case-histories of all these patients and further details of the urine analyses will be found in Appendices A and B.

### DISCUSSION.

Of particular interest in this case was the biochemical finding of heavy and prolonged ethanolaminuria. Clinically, also, the case was remarkable for the enormous size of the tumour. The liver weighed 13,120 g. and comprised almost a quarter of the body-weight (57,500 g.). Berman (1951) quotes 10,896 g. as the largest previously reported (in a case described by Cooper-Cole *et al.*, 1935).

*Pathogenesis of ethanolaminuria.*

The immediate cause of the ethanolaminuria was a raised plasma level for ethanolamine. The chromatograms showed this clearly, although the quantities involved were very small and the "spots" only just detectable. It has been shown that many of the known amino-acidurias result mainly, if not entirely, from a raised renal clearance (low threshold) for amino-acids (Dent, 1951*b*). It is not necessary to invoke this mechanism as a cause for the ethanolamine excretion here, since the normal subject who took ethanolamine by mouth produced a comparable urinary output at an induced blood level roughly similar to that of the patient. The increased ethanolamine excretion of the patient after an oral dose of 2·05 g. (i.e. 20 per cent of dose excreted in 24 hours) was not so great as the extra output of a normal subject after the same dose (i.e., 33 per cent of the dose in 24 hours). This paradoxical finding requires some explanation. We suggest that it can best be understood in terms of an increased membrane permeability, on the part of the tumour cells, for ethanolamine. If such abnormal permeability existed, a larger percentage of the loading dose of ethanolamine would pass from the extra-cellular fluid to the cells of the patient than it would in the case of the normal control. The control would, therefore, have a greater increase in plasma level, and would excrete a higher proportion of the test dose of ethanolamine than would the patient, at least in the first 24 hours. It is possible that, had the investigation been prolonged over a 3-day period, for the control as well as the patient, the percentages of the loading dose of ethanolamine excreted by both would have been seen to be the same. Reference to Fig. 9 shows clearly that in the case of the patient there was an increased output

FIG. 9.—Mrs. C. L—. Output of "apparent ethanolamine" on various dietary supplements.

of ethanolamine for 2 and probably 3 days after the test dose, as if it were still escaping from the tumour cells and becoming available for excretion by the kidney. Furthermore, this theory of altered membrane permeability would explain the chronically raised plasma level and, consequently, the markedly increased and continuous urinary excretion of ethanolamine in terms of the free escape of ethanolamine from the great bulk of the tumour, where it was being continuously synthesised.

If this explanation is correct this patient illustrates the type of defect previously described by one of us as a " deviation of metabolism " (Dent, 1950), and is not an " error of metabolism " in the sense used by Garrod (1923) with reference to certain congenital diseases.

*Diagnostic value.*

It is improbable that the findings of ethanolaminuria will prove to be important in the diagnosis of malignant disease of the liver. In the present series, besides the case under review, 7 other cases of primary carcinoma of the liver have been investigated, and in none was a similar isolated urinary excretion of ethanolamine found. It must be admitted, however, that none of these appeared to be related clinically or pathologically to the present case (Appendix A). The urine from 7 cases of extensive secondary carcinoma of the liver (Appendix B) was also investigated with negative results. It is not clear from the evidence at present available whether there is a specific, though rare, type of hepatic neoplasm characterised by an increased urinary excretion of ethanolamine or whether any tumour, if large enough, would give rise to a similar finding. The detection of ethanolaminuria in association with an increased excretion of certain other amino-acids, referred to earlier (Dent and Walshe, 1951), is probably a highly specific indication of liver damage; however, the finding in the present case (C. L—) is so far unique.

*Biochemical significance.*

The biochemical significance of the ethanolamine cannot, as yet, be assessed. Originally it was hoped to demonstrate a metabolic block in the first methylation stage (A) in the synthesis of choline, since this would readily account for the raised plasma and urine contents of ethanolamine, e.g. :

$$\begin{array}{ccccccc} \text{(A.)} & & \text{(B.)} & & \text{(C.)} & & \text{(D.)} \\ CH_2NH_2 + CH_3 & & CH_2NH \cdot CH_3 + CH_3 & & CH_2N(CH_3)_2 + CH_3 & & CH_2\overset{+}{N}(CH_3)_3 \\ | & \longrightarrow & | & \longrightarrow & | & \longrightarrow & | \\ CH_2OH & & CH_2OH & & CH_2OH & & CH_2OH \\ \text{(ethanolamine).} & & & & & & \text{(choline).} \end{array}$$

But, as has been mentioned already, we now prefer the theory of an increased membrane permeability for ethanolamine. The principal objection, perhaps, to the theory of a metabolic block is that if it were appreciable in extent it should have resulted in some degree of choline deficiency as well as in the accumulation of unmetabolisable ethanolamine. In experimental animals choline deficiency leads to fatty infiltration of the liver followed by fibrosis and, if sufficiently prolonged, to the development of hepatoma-like tumours (Copeland and Salmon, 1946 ; Staub, Viollier and Werthmann, 1948). It appeared possible, at one time, that our patient (C. L—) might have passed through these stages in acquiring her hepatoma. However, both needle biopsy of the liver in 1947 and the post-mortem examination in 1950 failed to reveal the presence of fatty change or of fibrosis of the liver. Moreover, the daily excretion of choline in the urine was within normal limits. Finally, the tumour sections were not at all similar to any of those, very kindly lent to us by Dr. Copeland, taken from rats which had been on a prolonged choline-deficient diet (Fig. 8 and 10). We are, therefore, unable to accept the possibility that the large urinary excretion of ethanolamine resulted from a disturbance of the known metabolism of choline or one of its important intermediate metabolites. We prefer to believe that it was caused by some other property of this particular type of heptoma, such as a disturbance of " cell membrane permeability."

A further problem has been posed by the complete failure to increase the excretion of ethanolamine in the experiments in which large doses of its known biochemical precursors (glycine and serine) were given by mouth. This fact appears to underline further the difficulties encountered in attempting to interpret the results of this type of " classical " experiment. Presumably the compounds failed to reach the cellular site where they would have been converted into ethanolamine and, therefore, that our patient did not provide a suitable opportunity to study normal ethanolamine metabolism by the rather crude methods at out disposal. The prospect of success would have been much greater using isotopically labelled precursors, since isolation from urine of pure samples of ethanolamine for isotype analysis presents no great difficulty.

In conclusion we wish to thank Sir Harold Himsworth for permission to publish this case, Professor M. L. Rosenheim for advice during the preparation of the manuscript, and Dr. D. H. Copeland for his very great kindness in lending us his original blocks of rat hepatoma tissue ; also the following for permission to investigate the cases referrred to in the appendices : Mr. A. J. Gardham, Dr. J. C. Hawksley, Dr. Gwen Hilton, Sir Harold Himsworth, Dr. J. D. Nabarro, Professor M. L, Rosenheim, Dr. Sheila Sherlock, Dr. J. F. Stokes, Dr. R. Terry and Mr. H. R. I. Wolfe. We also wish to thank Miss D. I. Fowler for carrying out the urinary ethanolamine estimations, Mr. J. Bligh for the choline assays and Mr. A. Bligh for the photomicrographs.

### SUMMARY.

1. A case of primary carcinoma of the liver in a woman who died at the age of 45 is described. The liver weighed 13,120 g. and, apart from effects due to its size, it was associated with fairly good health, and with normal liver function as determined by laboratory tests and by clinical assessment.

2. The urine of this patient was analysed for amino-acids on many occasions during the last 7 months of her life. It contantly showed on the paper chromatograms a large spot of ninhydrin-reacting material that was identified as ethanolamine. Otherwise the chromatograms showed no gross abnormality. This finding has not been encountered before either in normal or in pathological urines, these having included urine from very many cases of liver disease. A smaller but still excessive output of ethanolamine in the presence of increased outputs of certain other amino-acids is, however, commonly found in liver disease.

3. A chemical method of analysis suggested that the output of ethanolamine was of the order of 0·5 to 1·0 g. daily. This was confirmed by a rough quantitative chromatographic assay. The choline output, estimated by biological assay, was normal.

4. Preliminary studies were carried out to determine the metabolic origin of the ethanolamine and the mechanism by which such large quantities came to be excreted in the urine.

5. Our tentative explanation of the metabolic defect is as follows: The urinary excretion of ethanolamine resulted from a raised blood level. As this substance is probably not readily taken up even in normals from the extracellular fluids by the tissue cells or from the glomerular filtrate by the renal tubule cells, it was therefore readily excreted into the urine. The presumed source of the ethanolamine was the neoplastic liver cell, and the enormous mass of these cells (about a quarter of her body weight) in the patient was probably sufficient to account for a greatly excessive production of this compound. We further have to assume that the neoplastic cells were more "permeable" than normal, and that they allowed some of the ethanolamine formed in them to leak into the extracellular fluids and thence into the urine.

6. Seven other cases of primary and 7 of extensive secondary carcinoma of the liver have also been investigated. They all had much smaller livers than the patient described here in detail. None of them showed a comparable excretion of ethanolamine occurring in the absence of other amino-acid abnormalities.

### APPENDIX A.

### *Primary Carcinoma of the Liver: Case Reports.*

CASE 1.—J. R—, male, aged 44 years, a chronic alcoholic, died in hepatic coma within 24 hours of admission to hospital. Post-mortem examination revealed a cirrhotic liver, weight 3600 g.; there was extensive neoplastic change involving principally the left lobe; there were bile-forming secondaries in both lungs. Chromatographic analysis of the urine revealed a moderate generalised amino-aciduria, as is often found in severe hepatitis.

CASE 2.—G. K—, an Indian, aged 60 years. He had a cholecystectomy for gall stones in 1942. In 1950 he had an attack of renal colic and a ureteric calculus was demonstrated. In 1951 he was admitted to hospital with severe upper abdominal and left shoulder pain. Laparotomy revealed a moderate enlargement of the liver, with very numerous white opaque nodules scattered throughout the liver substance. A biopsy of one of these nodules showed a typical liver-cell carcinoma arising on a basis of cirrhosis. Chromatographic analysis of the urine showed a normal amino-acid concentration and pattern except for a slight excess of cystine and lysine.

CASE 3.—E. C—, male, aged 34 years. An adult case of the Fanconi syndrome, the details of which have been published elsewhere (Stowers and Dent, 1947). Liver weight 1500 g.; it showed well-marked carcinomatous change secondary to post necrotic scarring. Chromatographic analysis of the urine showed the heavy amino-aciduria typical of the Fanconi syndrone; no ethanolamine was detected.

CASE 4.—A. G—, male, aged 54 years. Details of this case have been published elsewhere (Flynn and Walshe, 1951). The liver was not enlarged or cirrhotic, there were two small nodules of liver-cell carcinoma in the left lobe. Chromatographic analysis of the urine revealed no abnormality in the concentration or pattern of amino-acids.

CASE 5.—B. C—, female, aged 14 years. She complained of abdominal swelling for 14 months. On examination, 8 months before admission to hospital, she was found to have an enlarged liver and spleen ; at this stage all liver function tests were negative and a urine amino-acid chromatogram showed only a slight excess of cystine. Four months later she complained of abdominal pain and laparotomy was performed ; this revealed a mass the size of a grape fruit in the right lobe of the liver which, on histological examination, proved to be an anaplastic carcinoma of the liver. There was no evidence of cirrhosis. She died a few weeks later. Post-mortem examination confirmed the diagnosis : the liver weighed 2930 g. Urine analysis a few days before death showed a slight but definite excessive output of cystine, β-amino-isobutyric acid and ethanolamine.

CASE 6.—G. H—, male, aged 71 years, was in good health until 2 weeks before admission to hospital when he had a severe attack of right upper abdominal pain lasting 1½ hours. During the ensuing fortnight he had several further attacks. Physical examination revealed no abnormality except a tender liver enlarged to below the umbilicus. Radiographic examination of the chest, kidneys and gastro-intestinal tract revealed no evidence of an extra-hepatic primary growth. Needle biopsy of the liver showed an undifferentiated carcinoma, possibly primary, in the liver. Chromatographic analysis of the urine for amino-acids showed a slight but definite excess of cystine and taurine.

CASE 7.—T. T—, male, aged 50 years. His complaint, on admission, was of right-sided chest pain. Examination showed him to have a hard enlarged liver and a palpable spleen. Liver function tests were normal except for a plasma alkaline phosphatase of 36 King units and a serum bilirubin of 2·1 mg. per 100 ml. Needle biopsy of the liver revealed a malignant hepatoma. This diagnosis was confirmed at post-mortem a few weeks later. Chromatographic analysis of the urine for amino-acids showed only a slight excess of cystine and β-amino-isobutyric acid.

### APPENDIX B.

### *Secondary Carcinoma of the Liver : Case Reports.*

CASE 1.—P. A—, male, aged 61 years, admitted to hospital with a 5 months' history of pruritis and progressive jaundice. He refused laparotomy, and died 6 weeks later. At post-mortem examination he was found to have a primary carcinoma of the extra-hepatic bile duct ; there was extensive secondary involvement of the liver, which weighed 4720 g. Chromatographic analysis of the urine showed a completely normal pattern of amino-acids until the day on which the patient died, when there was a heavy, generalised amino-aciduria compatible with an episode of terminal hepatic failure.

CASE 2.—R. C—, female, aged 75 years, was suffering from 1 month's right-sided upper abdominal pain, anorexia and nausea, and jaundice for 1 week. Physical examination revealed light jaundice, a right-sided pleural effusion, moderate irregular hepatic enlargement and a number of secondary carcinomatous nodules in the skin. She lapsed into coma, and died 4 days after admission to hospital. At post-mortem examination she was found to have a primary carcinoma of the head of the pancreas with extensive secondary involvement of the liver, which weighed 1760 g. Chromatographic analysis of the urine for amino-acids showed only a slightly raised concentration of cystine.

CASE 3.—L—, male, aged 60 years, formerly a heavy alcoholic, complained of 4 months' right subcostal pain and swelling of the abdomen. He was found, on examination, to have an enormous enlargement of the liver extending from the fourth intercostal space to the pelvic brim. Needle biopsy of the liver showed a secondary carcinoma. Urinary amino-acid excretion was normal except for a definite excess of cystine.

CASE 4.—, M—, male, aged 45 years. In 1945 he had an orchidectomy performed for the removal of a seminoma. In 1949 and 1950 he received three courses of deep x rays to his abdominal glands. In January, 1951, he became jaundiced and feverish. At that time his liver was enlarged four fingers' breadth below the right costal margin. Urine for chromatographic analysis was taken during the course of deep x-ray therapy to the liver ; the amino-acid pattern and concentration were normal except for a slight excess of cystine.

CASE 5.—A. P—, aged 40 years. He complained of 1 month's malaise and dysphagia and 2 days' jaundice. Examination revealed marked icterus, a liver enlarged almost to the umbilicus and a mass of carcinomatous glands in the left posterior triangle. His course was rapidly downhill, and at post-mortem examination he was found to have a bronchial carcinoma with secondaries in the mediastinum, liver and cervical glands. The liver weight was 2850 g. Chromatographic analysis of the urine for amino-acids showed a definite slight excess of cystine.

CASE 6.—J. H—, aged 53 years. Apart from a cholecystectomy in 1950 he was in good health until shortly before admission to hospital when he was seized with a severe low back pain, greatly aggravated by movement. On examination he was found to have extreme tenderness over the lumbar spine, reduced air entry at the left lung base and a liver enlarged below the umbilicus. A diagnosis was made of carcinoma of the bronchus with secondaries in the lumbar spine and liver. His course was marked by very rapid enlargement of the liver and progressive cachexia. Post-mortem examination was not permitted. Chromatographic analysis of the urine showed that the amino-acid concentration and pattern were normal.

CASE 7.—W. D—, male, aged 42 years, originally admitted for investigation of hepato-splenomegaly in 1950. Liver biopsy revealed a diagnosis of malignant lymphoma. By January, 1951, he was needing frequent abdominal paracenteses and his condition deteriorated rapidly. At post-mortem examination his liver was found to be almost entirely replaced by lymphomatous tissue, it weighed 2120 g. Chromatographic analysis of the urine for amino-acids showed that there were slightly excessive concentrations of cystine and ethanolamine and a large excess of taurine.

### REFERENCES.

BERMAN, C.—(1951) 'Primary Carcinoma of the Liver.' London (Lewis).
BLIGH, J.—(1952) *J. Physiol.*, **117**, 234.
BORGLIN, N. E.—(1947) *Acta pharm. tox., Kbh.*, **3**, Suppl. No. 1, 123.
BUCKLY, J. J., BUCKLY, S. M., AND SNIPES, A. E.—(1951) *Johns Hopk. Hosp. Bull.*, **89**, 218.
COOPER-COLE, C. E., ROGERS, S. O., NORWICH, A. C., AND LOUGHEED, G. W.—(1935) *Canad. med. Ass. J.*, **32**, 420
COPELAND, D. H., AND SALMON, W. D.—(1946) *Amer. J. Path.*, **22**, 1059.

Crumpler, H. R., and Dent, C. E. (1949) *Nature*, **164**, 441.
Dent, C. E.—(1950) *Schweiz. med. Wschr.*, **80**, 752.—(1951*a*) in 'Recent Advances in Clinical Pathology,' 2nd Edn. London (Churchill).—(1951*b*) 'Exposés Annuels de Biochimie Médicale.' Paris (Masson).
*Idem*, Fowler, D. I., and Walshe, J. M.—(1951) *Biochem. J.*, **48** xiii.
*Idem* and Walshe, J. M.—(1951) 'Liver Disease.' London (Churchill).
Flynn, F. V., and Walshe, J. M.—(1951) *Brit. med. J.*, **1**, 1484.
Garrod, A. E.—(1923) 'Inborn Errors of Metabolism', 2nd. Edn. Oxford Medical Publications.
Opie, E. L.—(1944) *J. exp. Med.*, **80**, 219.
Staub, H., Viollier, G., and Werthemann, A.—(1948) *Experientia*, **4**, 233.
Stowers, J. M., and Dent, C. E.—(1947) *Quart. J. Med.*, **16**, 275.